# Metal-based nanoplatforms for enhancing the biomedical applications of berberine: current progress and future directions

**DOI:** 10.1080/17435889.2025.2480051

**Published:** 2025-03-20

**Authors:** Isaac Baidoo, Paromita Sarbadhikary, Heidi Abrahamse, Blassan P. George

**Affiliations:** Laser Research Centre, Faculty of Health Sciences, University of Johannesburg, Johannesburg, South Africa

**Keywords:** Berberine, drug delivery, drug targeting, metallic nanoparticles, nanomedicine, natural products

## Abstract

The isoquinoline alkaloid berberine, a bioactive compound derived from various plants, has demonstrated extensive therapeutic potential. However, its clinical application is hindered by poor water solubility, low bioavailability, rapid metabolism, and insufficient targeting. Metal-based nanoplatforms offer promising solutions, enhancing drug stability, controlled release, and targeted delivery. This review comprehensively explores the synthesis, physicochemical properties, and biomedical applications of metal-based nanocarriers, including gold, silver, iron oxide, zinc oxide, selenium, and magnetic nanoparticles, for berberine delivery to improve berberine’s therapeutic efficacy. Recent advancements in metal-based nanocarrier systems have significantly improved berberine delivery by enhancing cellular uptake, extending circulation time, and enabling site-specific targeting. However, metal-based nanoplatforms encounter several limitations of potential toxicity, limited large-scale productions, and regulatory constraints. Addressing these limitations necessitates extensive studies on biocompatibility, long-term safety, and clinical translation. By summarizing the latest innovations and clinical perspectives, this review aims to guide future research toward optimizing berberine-based nanomedicine for improved therapeutic efficacy.

## GRAPHICAL ABSTRACT

**Figure:**
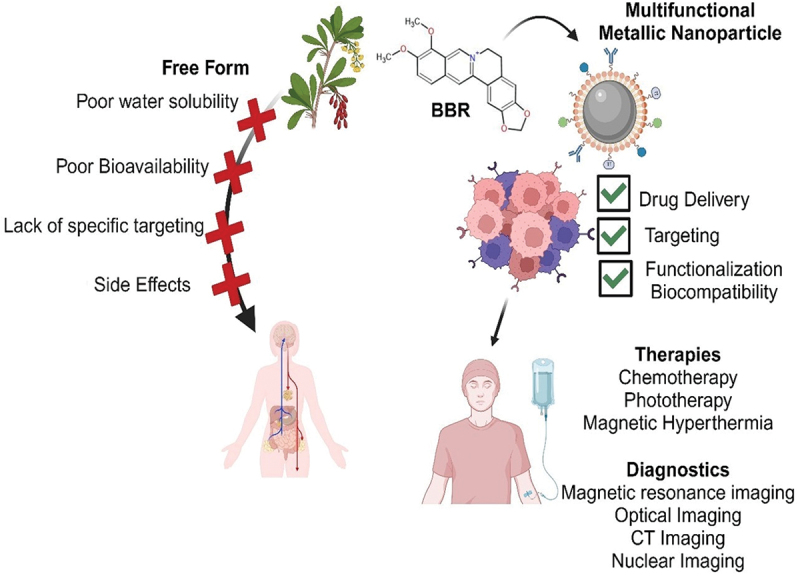

## 1. Introduction

As per a report published by the World Health Organization, it has been noted that 80% of the populace in developing nations relies on traditional medicine for their primary healthcare needs [1]. This reliance has instigated rigorous scientific inquiries into the chemical and pharmacological attributes of plants to formulate bioactive compounds that exhibit heightened efficacy and reduced side effects [2]. Drug development from natural products has notable advantages, including lower toxicity, reduced side effects, affordability, and substantial therapeutic potential. Nonetheless, identifying active compounds from natural sources most especially plants poses difficulties due to changes in living organisms’ metabolite composition under various conditions and issues with solubility, stability in aqueous solutions, safety, and regulatory approval [3].

**Figure 1.**
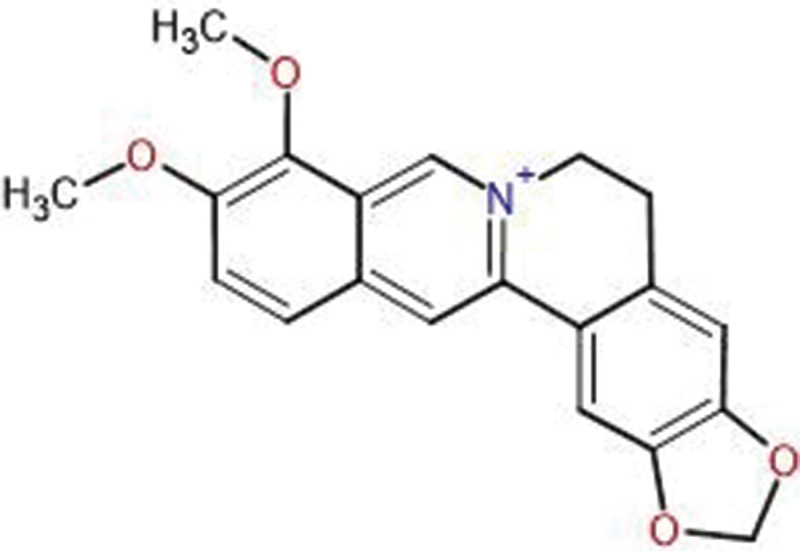
The chemical structure of BBR. The structure features a quaternary ammonium group and several rings, both of which play a crucial role in its biological functions. The planar, positively charged structure of BBR enables it to bind with nucleic acids and interact with proteins, leading to a range of pharmacological activities, such as anticancer, antimicrobial, and anti-inflammatory effects.

Berberine (BBR), a natural isoquinoline alkaloid (Figure 1) derived from various plants such as *Berberis aristata*, *Berberis vulgaris*, *Coptis chinensis*, and *Hydrastis canadensis*, has long been recognized for its multifaceted pharmacological activities. It displays a wide range of biological activities [4], including antimicrobial [5], antiviral [6], antidiarrheal [7], antipyretic [8], and anti-inflammatory activities [9]. However, BBR’s clinical use is limited by poor water solubility, resulting in a bioavailability of only around 5% [10,11]. However, the clinical utility of BBR is significantly hindered by its poor aqueous solubility, low bioavailability, low gastrointestinal absorption, high plasma protein binding and rapid systemic elimination [12]. To improve its therapeutic effectiveness, nanoparticle-based delivery systems are being developed to enhance BBR’s efficacy [13]. Nanocarrier systems such as polymeric nanoparticles, lipid-based nanoparticles, and metal-based nanoplatforms offer different advantages for drug delivery [14]. Polymeric nanoparticles (e.g., PLGA, chitosan) provide biocompatibility, controlled drug release, and ease of functionalisation [15]. However, they often suffer from batch-to-batch variability, degradation issues, and potential immune responses [15]. Lipid-based carriers (e.g., liposomes, solid lipid nanoparticles) enhance solubility and bioavailability but can be unstable in circulation and prone to rapid clearance [16]. Metal-based nanoplatforms, on the other hand, offer unique advantages: (i) enhanced stability in biological environments [17], (ii) tunable surface properties for functionalisation [17], (iii) superior drug loading capacity [18], (iv) targeted delivery via magnetic or plasmonic properties [19], and (v) potential for combination therapies (e.g., photothermal and photodynamic therapy) [20]. Despite these advantages, metal nanoparticles also face challenges related to toxicity, biodistribution, and long-term safety, which require further optimization through green synthesis and surface modifications.

Research focusing on various nanoparticle systems for the delivery of BBR has seen significant expansion, particularly in the field of metallic nanoparticles. These nanoparticles play vital roles in bioimaging, biosensors, targeted drug delivery, as well as hyperthermia and photoablation therapies [21]. They are engineered and functionalized with specific groups to bind to antibodies, drugs, and other biological agents, thereby increasing their biomedical potential [22]. Nanoconjugates of BBR with metal nanoparticles, such as gold, silver, iron oxide, and zinc oxide, present a novel paradigm for enhancing the therapeutic efficacy and versatility of this bioactive compound. These hybrid systems not only improve the solubility and stability of BBR but also facilitate targeted delivery and controlled release, thereby maximizing its therapeutic benefits while minimizing systemic toxicity [23]. Furthermore, the inherent properties of metal nanoparticles offer the advantages of synergizing with BBR’s therapeutic potency, enhancing its bioactivity and enabling new functionalities, such as photothermal therapy, magnetic resonance imaging, and antibacterial coatings. In addition to the well-studied nanoparticles made of gold, silver, iron, and copper, growing interest is directed toward other metals such as zinc oxide, titanium oxide, platinum, selenium, gadolinium, palladium, and cerium dioxide due to their unique properties and potential in medical applications [24,25].

This review emphasizes the critical role of metal nanoparticles as carriers for BBR in medical settings. It provides insight into the synthesis, therapeutic and biomedical applications of metal-based nanoplatforms for BBR delivery, aiming to guide researchers and healthcare professionals in using these advanced systems for targeted therapeutic strategies and unlocking new therapeutic avenues.

## 2. Therapeutic application of BBR

BBR demonstrates a wide range of pharmacological activities, traditionally utilized for its antimicrobial, antiprotozoal, and antidiarrheal properties. Modern research has expanded its therapeutic potential, revealing promising effects in cancer treatment, diabetes management, central nervous system disorders, cardiovascular health, and anti-inflammatory applications. These findings highlight BBR’s versatility as a bioactive compound in both traditional and contemporary medicine [26]. Consuming BBR as a standalone is generally considered safe for most adults; however, some individuals may experience side effects, primarily affecting the digestive system. Common adverse effects include gastrointestinal Issues (diarrhea, constipation, gas, or an upset stomach) [27], abdominal Discomfort (pain or cramping) [28], nausea [29] and headaches [30]. In rare instances, BBR can cause a significant drop in blood pressure, leading to dizziness or lightheadedness [31].

**Figure 2.**
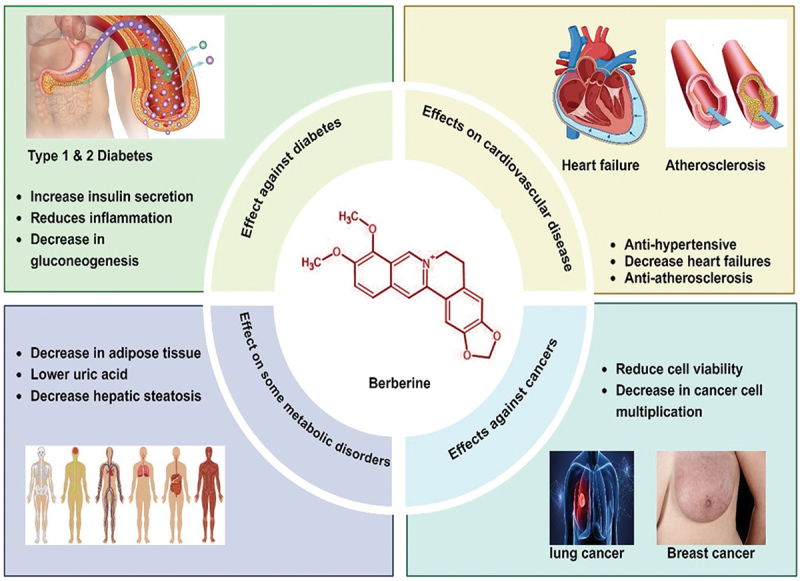
Potential role of BBR in different health conditions. This figure highlights the various therapeutic applications of BBR in a range of health conditions. BBR has been shown to help manage metabolic issues, including obesity, diabetes, and hyperlipidemia, by enhancing insulin sensitivity and regulating lipid levels. It also supports cardiovascular health by lowering inflammation and cholesterol. In addition, BBR provides neuroprotective benefits in disorders such as Alzheimer’s and Parkinson’s diseases, while also offering anti-inflammatory, antimicrobial, and anticancer properties. These diverse effects underscore BBR’s potential as a multi-functional natural remedy. created with BioRender.com.

This section provides an analysis of the pivotal role and molecular mechanism of BBR as a highly promising pharmaceutical candidate for the management of various conditions, including cancer, diabetes, hyperlipidemia, and others, as depicted in Figure 2.

### 2.1. Anticancer effect

BBR is a potential compound derived from various plants that has been used in cancer treatment and has shown significant efficacy against various cancers, including breast, lung, cervical, and gastric cancers [1].

BBR has been shown to reduce cell viability and inhibit cancer cell growth. It also decreases levels of metadherin, a protein associated with breast cancer cell spread, thereby limiting proliferation [32]. BBR regulates cellular processes by disrupting cyclin D/CDK4 complexes, which activate p38, a cell growth inhibitor, leading to cell cycle arrest at the G1/S phase. It further inhibits cancer cell growth by blocking the cyclin A/CDK1 kinase complex and disrupting the AKT/ERK signaling pathways, resulting in G2/M phase cell cycle arrest [32,33]. Additionally, BBR reduces abnormal cell growth and induces apoptosis. When combined with chemotherapy drugs like cisplatin, 5-fluorouracil, methyl methane sulfonate, and camptothecin, BBR can enhance cancer treatment efficacy [32,33]. Moreover, BBR lowers the expression of AMPK and HIF-1α proteins, making MFC-7/hypoxia cancer cells more sensitive to doxorubicin in low-oxygen conditions [34]. Studies show that BBR can inhibit the growth of non-small cell lung cancer (NSCLC) xenograft tumors by targeting the SWI-independent-3 transcription regulator. BBR induces DNA damage in NSCLC cells by reducing topoisomerase II (TOP2) levels, leading to cell death, while also activating the miR19a/TF/MAPK signaling pathway to further promote cell death [35,36]. Further, several key pathways are affected by BBR, including the induction of cell cycle arrest, apoptosis, autophagy, inhibition of cell invasion, modulation of microRNA expression, telomerase activity, and the tumor microenvironment, underscoring berberine’s therapeutic potential in lung cancer treatment [37].

Studies show that BBR boosts the effectiveness of various anticancer drugs in limiting ovarian cancer cell growth. Its synergistic action with cisplatin leads to cell inactivation, disrupts cellular processes and promotes programmed cell death through caspase-dependent and RIPK3-MLKL pathways [38]. Additionally, BBR inhibits the iPLA2-AA-COX-2-PGE2 pathway and counters the growth-promoting effects of FAK phosphorylation triggered by the chemotherapeutic agent VP16 [39]. Combining BBR with niraparib offers a strategic approach to combatting ovarian cancer by causing DNA damage [40]. Zhao et al. explored the role of berberine in alleviating chemotherapy-induced side effects [39]. Their research demonstrated that chemotherapy accelerates metastasis by up-regulating the transcription factor GLI1, which subsequently increases the expression of the pluripotency gene BMI1 and epithelial-mesenchymal transition (EMT) markers, including Vimentin and Snail. BBR was shown to counter these effects by reducing cancer stem cell-like properties and reversing EMT and migration through the inhibition of the GLI1/BMI1 signaling pathway, which chemotherapy activates. These findings indicate that BBR may serve as an effective adjuvant therapy, potentially preventing chemotherapy-induced metastasis in ovarian cancer by targeting key molecular pathways [38,39]. Research has also shown that BBR is effective against stomach cancer through various mechanisms. Berberine induces G1 phase cell cycle arrest, restricting the proliferation of SGC-7901 cells [41]. Additionally, it has been found to inhibit cancer cell growth and reduce IL-8 secretion by deactivating MAPK signaling pathways, both in vitro and in vivo [42]. BBR also combats stomach cancer by inhibiting STAT3 activation and reducing the phosphorylation of the epidermal growth factor receptor (EGFR) [41,43].

**Figure 3.**
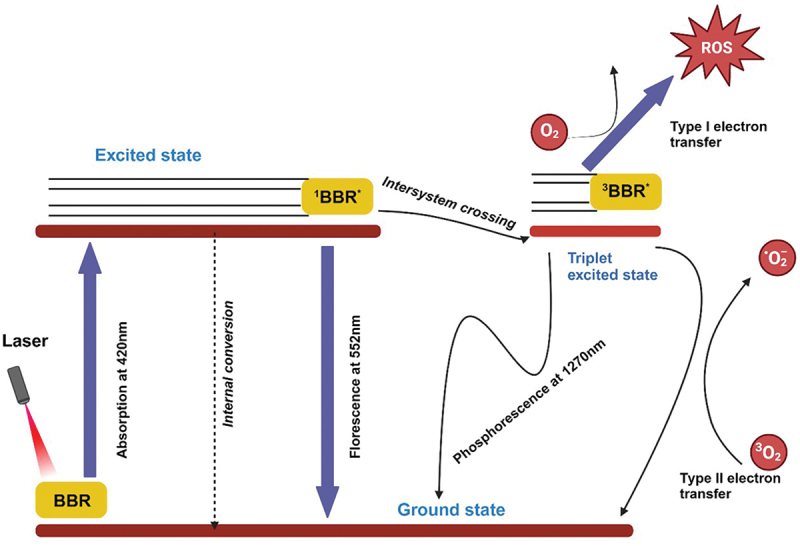
Photochemical reaction of BBR. This figure depicts the photochemical reaction process of BBR. When exposed to light, BBR transitions to an excited state, triggering the production of ROS, such as singlet oxygen and free radicals. These ROS are crucial in PDT as they induce oxidative stress in targeted cells, leading to cell damage and apoptosis. This reaction underscores BBR’s potential as an effective photosensitizer in cancer treatment approaches. created with BioRender.com.

Other than its anticancer potential, BBR also shows photoactive potential akin to some other plant-derived substances [44–46]. Moreover, BBR exhibits a notable affinity for low-density lipoproteins (LDL), thereby facilitating cellular uptake and offering the prospect of targeted delivery to tumor cells [47]. Andreazza et al. conducted a study investigating BBR’s association with LDL and its subsequent effects on accumulation and phototoxicity in glioma cells, results showed that delivering BBR via LDL enhanced its photo-cytotoxic effects on glioma cells [47]. As a result, researchers have sought to investigate the potential of BBR as a photosensitizer PS in PDT for cancer treatment (Figure 3).

The study by Wang and colleagues revealed that BBR-mediated PDT enhanced the effectiveness of cisplatin in cisplatin-resistant melanoma cells by activating the ROS/p38/caspase cascade [48]. Oliveira et al. showed the decrease in cell viability to 20% in carcinoma cells and 47% in keratinocytes. The increase in reactive oxygen species (ROS) production and caspase-3 activity suggested apoptosis through a caspase-dependent pathway [48]. Similarly, another study on BBR-mediated PDT for renal cancer cell lines showed the increased ROS levels responsible for enhanced cell death through both autophagy and apoptosis via caspase-3 activity [49]. Antiproliferative effects of BBR-PDT on malignant melanoma cells is attributed to apoptosis induction via upregulation of cleaved caspase-3 and autophagy activation through increased LC3 expression [50]. BBR induced strong phototoxicity in human astrocytoma resulting into significant apoptosis induction, ROS production, mitochondrial depolarization, and caspase activation [51].

Mechanistically, several studies have shown that BBR can induce phototoxicity by directly binding to DNA leading to DNA cleavage through^1^ O₂-mediated photooxidation [52,53]. Further, BBR can also enhance DNA cleavage in neutral aqueous solutions by reacting with hydrated electrons and hydroxyl radicals, producing BBR radical species [54]. BBR’s absorption spectrum is characterized by distinct peaks between 350 nm and 450 nm. However, the clinical applicability of the 450 nm wavelength is limited by its reduced tissue penetration, while the presence of melanin exerts antioxidant effects that diminish the efficacy of PDT in cutaneous applications [55]. The effective harnessing of BBR’s properties requires the adoption of advanced formulations, encompassing drug combinations and delivery systems.

### 2.2. Metabolic disorders

BBR has attracted considerable interest for its role in treating various conditions, such as Type 2 Diabetes Mellitus (T2DM), Non-Alcoholic Fatty Liver Disease (NAFLD), and hyperlipidemia [56]. Table 1 below provides a comprehensive summary of key findings from scientific studies assessing BBR’s effectiveness in managing these metabolic disorders.

**Table 1.** Overview of BBR efficacy in metabolic disorders and related conditions.

Metabolic Disorder	Associated Conditions	BBR Effects	Key Studies
Obesity	Increases risk of T2DM, NAFLD, hypertension, hypercholesterolemia, cancer	BBR improves glycemic control (reduces HbA1c, FPG, 2hPG), enhances insulin sensitivity, and lipid profiles, and reduces inflammation	[57–62]
NAFLD	Linked to obesity, insulin resistance, dyslipidemia	BBR modulates lipid/glucose metabolism, reduces inflammation and oxidative stress, improves liver fat content and enzyme levels	[63–65]
Hyperlipidemia	High cholesterol, triglycerides, LDL-C	BBR reduces TC, TG, and LDL-C levels and improves liver function; more effective than some statins	[59,60,66–68]
Gout	Hyperuricemia, increased risk of metabolic disorders	BBR reduces serum uric acid levels, combined with other treatments, improves metabolic disorders	[69–72]
Polycystic Ovary Syndrome (PCOS)	Insulin resistance, ovulatory dysfunction	BBR improves insulin resistance, enhances ovulation, improves lipid profiles, and increases pregnancy rates	[57,73–77]
Cardiovascular Diseases (CVD)	Atherosclerosis, hypertension, heart failure	BBR reduces cholesterol, inflammation, and vascular stiffness, improves blood pressure control, and supports heart function	[32,45,66,71,76,78–85]
Neuropathy	Alzheimer’s, Parkinson’s, Huntington’s	BBR shows neuroprotective effects, improves memory, reduces neurotoxicity and neuronal degeneration	[84–92]

This table summarizes the effects of BBR on various metabolic disorders and their associated conditions. BBR has demonstrated significant benefits in managing obesity, improving blood sugar control, insulin sensitivity, and lipid profiles, while reducing inflammation. In NAFLD, BBR helps regulate lipid and glucose metabolism, lowers inflammation, and enhances liver health. For hyperlipidemia, BBR effectively reduces cholesterol and triglyceride levels, with efficacy comparable to some statin medications. In gout, BBR lowers serum uric acid levels and improves metabolic health, especially when used with other treatments. It also improves insulin resistance and ovulation in women with polycystic ovary syndrome (PCOS). Additionally, BBR shows promise in cardiovascular health by lowering cholesterol, reducing inflammation, and supporting heart function. In neurodegenerative disorders like Alzheimer’s and Parkinson’s, BBR has neuroprotective properties, aiding in memory enhancement and reducing neuronal damage.

### 2.3. Anti-diabetic properties

Recent preclinical and clinical studies have shown BBR’s significant effects on lipid and carbohydrate regulation, particularly in controlling glucose levels [45]. BBR enhances insulin receptor performance in the liver and skeletal muscles by activating kinase C-dependent proteins [93]. In a study by Yin et al., BBR effectively managed type 2 diabetes, consistently lowering fasting and post-meal glucose levels [94]. The study also reported a 28.1% reduction in fasting plasma insulin and a 44.7% decrease in the HOMA-IR index (*p* < 0.001), along with a drop in hemoglobin A1C levels from 8.1% to 7.3% (*p* < 0.001). While 34.5% of participants experienced temporary gastrointestinal discomfort, no adverse effects on liver or kidney function were observed. These results highlight BBR’s potent hypoglycemic effects and its positive impact on lipid metabolism, positioning it as a promising treatment for diabetes [95].

Kong and colleagues studied the cellular mechanisms behind BBR’s enhancement of insulin sensitivity, finding that BBR stimulates the insulin receptor (InsR) [36,95,96]. Their research showed that BBR treatment increased InsR expression in a dose- and time-dependent manner at both mRNA and protein levels in human liver cells. In diabetic mice with type 2 diabetes, BBR significantly lowered blood glucose levels, though this effect was not seen in non-obese mice with type 1 diabetes [36,95,96]. These findings highlight BBR’s potential as a promising phytochemical for treating insulin resistance in type 2 diabetes.

### 2.4. Antiviral activities

BBR has emerged as a potent broad-spectrum antiviral agent. A study by Giannone et al. (2023) demonstrated that BBR possesses broad-spectrum inhibitory activity against various strains of Zika virus (ZIKV) and all four serotypes of dengue virus (DENV) in both Vero cells and human cell lines [97]. The mechanisms underlying BBR’s antiviral properties were shown to inhibit the activation of ERK1/2 and p38 signaling pathways triggered by ZIKV. Additionally, BBR was found to reduce p38 phosphorylation in uninfected cells, suggesting its antiviral effects may be closely tied to the modulation of p38 signaling [97]. Further reinforcing these findings, earlier research by Warowicka et al. showed that BBR exhibits antiviral activity against human cytomegalovirus by inhibiting viral DNA replication and decreasing the production of viral particles [92].

A study by Sekar et al. investigated the effectiveness of BBR in inhibiting HCV replication by reducing the expression of the HCV core protein and blocking the viral RNA polymerase [98]. BBR effectively inhibited the replication of the influenza A virus by disrupting the nuclear export of viral ribonucleoproteins [99]. BBR strongly inhibits H1N1 influenza A strains and shows antiviral activity against herpes simplex virus by reducing viral RNA transcription and protein synthesis [100]. Additionally, BBR is highly effective against the HIV-1 NL 4.3 virus [101,102].

BBR has also been shown to be effective against the severe acute respiratory syndrome coronavirus (SARS-CoV), which helps reduce the risk of acute lung injury (ALI) and acute respiratory distress syndrome (ARDS) in COVID-19 patients by suppressing the release of inflammatory cytokines and modulating key inflammatory pathways [103]. Warowicka et al. emphasized BBR’s broad antiviral effectiveness against several viruses, including coronaviruses, influenza, respiratory syncytial virus, and herpes viruses [92].

### 2.5. Antimicrobial activities

BBR is well known for its broad-spectrum antimicrobial properties. Notably, Sadeghi et al.‘s study demonstrated BBR’s robust antibacterial efficacy against methicillin-resistant *Staphylococcus aureus* (MRSA) by impeding bacterial cell membrane integrity and nucleic acid synthesis, resulting in a substantial decline in bacterial viability [104]. Similarly, Yang et al. reported that BBR is effective against *Helicobacter pylori* by inhibiting the urease activity of *H. pylori*, which is essential for the bacterium’s survival in the acidic environment of the stomach. Furthermore, berberine was found to disrupt the bacterial biofilm, enhancing the efficacy of conventional antibiotics [105]. BBR targets the bacterial cytokinesis protein Fts-Z, disrupting Z ring formation and interfering with the cytokinesis process, leading to the destabilization of Fts-Z protofilaments and inhibition of its GTPase activity [106].

A study by Wu et al. employed untargeted metabolomics to uncover new insights into the antimicrobial mechanism of BBR against *S. aureus*. The research revealed that BBR exerts its antibacterial effects by disrupting multiple metabolic pathways within the bacterium. Specifically, the study identified significant downregulation of pyridine dicarboxylic acids, which are essential for bacterial stress resistance [107]. Additionally, BBR exposure led to an accumulation of oxidized phospholipids and a reduction in critical lipid antioxidants such as gamma-tocopherol and farnesyl pyrophosphate, suggesting that BBR impairs the bacterial antioxidant defense system. Moreover, the inhibition of key metabolites involved in peptidoglycan synthesis, such as D-Ala-D-Ala, indicated that BBR effectively disrupts cell wall biosynthesis, a critical factor in maintaining bacterial cell integrity [107].

In addition to its antibacterial effects, BBR has demonstrated antifungal activity. A study by Lin et al. showed that BBR inhibits the growth of *Candida albicans* by inducing oxidative stress and damaging the fungal cell membrane [108]. BBR also shows antiparasitic effects against anaerobic protozoa such as *Giardia lamblia*, *Trichomonas vaginalis*, and *Entamoeba histolytica*. In cellular studies, it is effective against the dog roundworm *Toxocara canis* [109]. Additionally, when combined with the malaria drug pyrimethamine, BBR is more effective at eliminating infections compared to combinations like pyrimethamine with tetracycline or cotrimoxazole [110,111].

## 3. Limitations of BBR

Despite its promising therapeutic qualities, BBR’s pharmacokinetics and bioavailability limitations pose serious obstacles to clinical applications. These challenges have raised concerns about its therapeutic efficacy and have prompted extensive research to overcome these barriers.

One of the main challenges limiting the clinical use of BBR is its extremely low bioavailability, which arises from factors such as poor solubility, inefficient absorption in the gastrointestinal tract, rapid metabolism, and extensive elimination from the body [112]. Research indicates that less than 1% of orally administered BBR enters systemic circulation, making it difficult to reach therapeutic levels in the body [113]. Its low solubility in water hinders its dissolution in the gastrointestinal system, while its poor permeability through intestinal epithelial cells further restricts absorption into the bloodstream [64]. In addition, the first-pass metabolism in both the liver and intestinal wall significantly reduces the active form of BBR in circulation, exacerbating its bioavailability issues.

BBR’s pharmacokinetics also pose significant hurdles. When taken orally, it is rapidly broken down into inactive metabolites such as berberrubine, thalifendine, and demethyleneberberine by liver enzymes, particularly those belonging to the cytochrome P450 family [114]. This fast metabolism results in a short half-life, necessitating high or frequent dosing to maintain therapeutic levels, which increases the likelihood of toxicity and lowers patient adherence. Moreover, BBR undergoes extensive biliary excretion, further reducing its availability in the bloodstream [115].

Another major obstacle is the P-glycoprotein (P-gp) efflux mechanism, which actively transports BBR out of intestinal epithelial cells and back into the intestinal lumen, significantly impairing its absorption [116]. P-gp, an energy-dependent transporter, plays a key role in reducing the intracellular concentration of BBR, preventing it from reaching effective levels in the bloodstream. In cancer therapy, this efflux mechanism not only reduces berberine’s systemic bioavailability but also contributes to resistance in cancer cells, further limiting its therapeutic potential [12].

These limitations in BBR’s bioavailability and pharmacokinetics present serious challenges, particularly for clinical applications such as cancer therapy, where sustained therapeutic levels are necessary for effectiveness. The low systemic absorption and rapid elimination of BBR make it difficult for the compound to maintain its therapeutic activity in target tissues like tumors. As a result, higher doses are often required to compensate for its poor absorption, increasing the risk of adverse effects such as liver toxicity and gastrointestinal irritation, which further limit its practical use [113].

To overcome these issues, researchers have been investigating various strategies to improve berberine’s bioavailability and pharmacokinetic profile. Innovations include advanced drug delivery systems like nanoparticles, liposomes, and solid lipid nanoparticles, which enhance BBR’s solubility, stability, and delivery to specific targets [117]. Co-administering BBR with inhibitors of P-gp has also shown promise in reducing the efflux of the compound, thereby improving its absorption [116]. Additionally, chemical modifications of BBR’s molecular structure to improve its solubility and resistance to rapid metabolism are being studied as potential solutions [112]. These advancements offer hope for addressing the pharmacokinetic barriers that currently limit berberine’s application in clinical practice.

## 4. Nanoconjugates and bio-nanoconjugates of BBR

Nanoconjugates and bio-nanoconjugates of BBR have emerged as promising strategies to overcome the limitations associated with BBR’s pharmacokinetics and clinical applications, enhancing its therapeutic potential across various disease models [118]. These nanoscale delivery systems significantly improve the bioavailability, solubility, and stability of BBR, while also enabling targeted delivery and sustained drug release. Such advancements not only optimize the therapeutic effects of BBR but also minimize systemic toxicity, a critical challenge in its conventional formulations [119].

Nanoconjugates are synthetic carriers designed to encapsulate or conjugate BBR, employing delivery platforms such as polymeric, lipid-based, metal-based, and carbon-based nanoparticles [104,114]. Among these, polymeric nanoparticles constructed from biocompatible and biodegradable polymers, such as PLGA (poly (lactic-co-glycolic acid)) and chitosan, have demonstrated notable efficacy in encapsulating BBR [120,121]. These systems provide stability, prolonged circulation, and controlled drug release, making them ideal for long-term therapeutic applications. For example, Gupta et al. investigated polyamidoamine (PAMAM) dendrimers loaded with BBR for breast cancer treatment. Their results showed that PAMAM-BBR complexes significantly enhanced anticancer effects compared to free BBR, with covalent conjugation allowing higher drug payloads and better efficacy [122]. Lipid-based nanoparticles, including liposomes, solid lipid nanoparticles and nanostructured lipid carriers, also offer notable advantages, particularly in improving the solubility and controlled release of BBR [112,123]. These lipid systems reduce dosing frequency and minimize adverse effects, thereby enhancing patient compliance. Similarly, metal-based nanoparticles, such as gold and silver nanoparticles conjugated with BBR, have gained attention for their synergistic effects in cancer and microbial therapies, leveraging photothermal and photodynamic properties to improve therapeutic efficacy [124,125]. Furthermore, carbon-based carriers, such as graphene oxide and carbon nanotubes functionalized with BBR, enhance cellular uptake and cytotoxicity, particularly in cancer models [126].

Bio-nanoconjugates derived from biological and eco-friendly materials represent an innovative approach to drug delivery [127]. These systems are typically synthesized using green methods, utilizing biological resources such as plant extracts, microbial systems, or polysaccharides as reducing and stabilizing agents. This process not only eliminates the use of toxic chemicals but also enhances the biocompatibility of nanoparticles [128]. For instance, bio-nanoparticles synthesized from plant extracts, such as *Azadirachta indica*, offer added bioactivity due to the phytochemicals present in the extracts. These nanoparticles not only improve the stability and solubility of BBR but also contribute to its therapeutic effects through synergistic actions [117,129]. Similarly, microbial systems, including bacteria, fungi, and algae, have been employed to produce nanoparticles sustainably. These biologically derived nanoparticles, conjugated with BBR, exhibit superior anticancer and antimicrobial properties due to their high surface reactivity and biocompatibility [130]. The incorporation of phytochemicals and microbial derivatives in bio-nanoparticles enables multifunctional systems capable of addressing a wide spectrum of therapeutic needs, such as antioxidant, antimicrobial, and anti-inflammatory applications (Table 2) [136].

**Table 2.** Summary of various nanoconjugates and bio-nanoconjugates of BBR, highlighting their composition, functional properties, and potential biomedical applications.

Therapeutic Activity	Type of Nanocarrier	Example of Nanoconjugate	Mechanism/Advantage	Reference
Anticancer	Polymeric Nanoparticles	PLGA-BBR Nanoparticles	Enhanced cellular uptake, sustained drug release, and selective tumor targeting.	[117]
	Lipid Nanoparticles	Liposome-Encapsulated BBR	Improved solubility, bioavailability, and reduced systemic toxicity	[113]
	Metal Nanoparticles	Gold-BBR, Iron Oxide-BBR Nanoparticles	Synergistic photothermal and anticancer effects	[116]
	Carbon-Based Nanoparticles	BBR-Conjugated Graphene Oxide	Enhanced cellular penetration and induction of apoptosis in cancer cells	[64]
Antimicrobial	Metal Nanoparticles	Silver-BBR Nanoparticles	Synergistic antimicrobial effects against drug-resistant pathogens	[114]
	Lipid Nanoparticles	Solid Lipid Nanoparticles (SLNs) Loaded with BBR	Enhanced antibacterial activity and improved bioavailability	[115]
Antidiabetic	Polymeric Nanoparticles	Chitosan-BBR Nanoparticles	Sustained release for long-term glucose regulation	[112]
	Lipid Nanoparticles	NLCs Loaded with BBR	Improved absorption and enhanced therapeutic efficacy for managing diabetes.	[131,132]
Antioxidant	Biologically Derived Nanoparticles	Green-Synthesized (Plant-Based)	Enhanced antioxidant activity due to eco-friendly synthesis and high bioavailability	[133]
Antiparasitic	Biologically Derived Nanoparticles	Bovine serum albumin Nanoparticle with Berberine and albendazole	Enhanced bioavailability and intestinal absorption of BBR exploiting BBR’s antiparasitic property.	[134]
Anti-hyperlipidemia	Lipid Nanoparticles	BBR loaded in liposomes	BBR-loaded liposomes reduced total cholesterol, triglycerides and low-density lipoprotein cholesterol in hyperlipidemic mice.	[135]

The table highlights therapeutic applications of berberine (BBR) nanoconjugates, categorized by nanocarrier type, specific examples, mechanisms or advantages, and references. Nanocarriers include polymeric, lipid, metal, carbon-based, and biologically derived nanoparticles, with advantages such as enhanced bioavailability, sustained release, and synergistic effects.

One of the most notable applications of bio-nanoparticles is in cancer therapy. These nanoparticles, when loaded with BBR, demonstrate enhanced anticancer activity by improving their solubility, stability, and tumor-targeting efficiency [137]. For example, gold nanoparticles synthesized using green methods exhibit significant photothermal effects and cytotoxicity against cancer cells, enabling synergistic treatment in cancer therapy [138,139]. Silver bio-nanoparticles conjugated with BBR have also proven effective against multidrug-resistant pathogens, offering a synergistic antimicrobial effect by leveraging the properties of both silver and berberine [140]. Moreover, bio-nanoparticles have shown substantial promise in addressing biofilm formation, a major obstacle in chronic infections, thereby paving the way for innovative antimicrobial therapies [141].

In addition to their applications in cancer and antimicrobial treatments, bio-nanoparticles have been explored for their antioxidant and anti-inflammatory properties [142,143]. Green-synthesized nanoparticles retain the bioactivity of plant-based phytochemicals, enhancing BBR’s ability to neutralize ROS and mitigate oxidative stress. This makes them suitable for treating oxidative stress-related conditions, promoting tissue repair, and accelerating wound healing [144]. Furthermore, in managing diabetes, chitosan-based bio-nanoparticles loaded with BBR have demonstrated sustained drug release, enabled long-term glucose regulation and reduced the frequency of administration [145].

## 5. Metal nanoparticles for drug delivery and therapeutic applications

Metallic nanoparticles (MNPs) are increasingly applied in various medical fields, including bioimaging, biosensing, drug delivery, hyperthermia, and photoablation therapies [146,147]. By attaching functional groups, these nanoparticles can interact with antibodies, drugs, and other ligands, enhancing their biomedical applications. While gold, silver, iron, and copper nanoparticles have been well studied, growing attention is focused on alternative metals and metal oxides like zinc oxide, titanium oxide, platinum, selenium, gadolinium, palladium, and cerium dioxide for their unique properties [146–148].

MNPs such as silver, gold, palladium, titanium, zinc, and copper, possess adaptable optical properties. These nanoparticles can be functionalized to attach to targeting agents and active biomolecules via mechanisms like hydrogen bonding, covalent bonding, and electrostatic interactions. These modifications facilitate the incorporation of multiple drugs, thereby augmenting therapeutic effectiveness [4]. Magnetic nanoparticles serve to enhance the solubility of hydrophobic drugs in water, extend the circulation of drugs in the bloodstream, and impede their rapid excretion via the kidneys. Moreover, multifunctional nanoparticles can convey multiple bioactive agents and imaging agents, thereby enabling targeted delivery through surface ligand modification. The combination of cancer treatment with diagnostic capabilities marks a breakthrough in the field [4].

The primary objectives of drug delivery systems are to direct therapeutic agents to the targeted site, minimize side effects on healthy tissues, and regulate drug release to avoid over or under-dosing [5]. MNPs support these goals by optimizing surface coatings for controlled drug loading, delivery, and release at specific sites [6]. Effective drug delivery with MNPs requires designing them for gradual, sustained release and targeting drugs to specific areas without impacting surrounding healthy cells [7]. This can be accomplished through passive targeting, which exploits alterations in cancer vasculature, and active targeting, where MNPs are linked with active ligands to bind specific cellular receptors, enabling precise therapeutic delivery [149].

Magnetic nanoparticles are presently being examined in both early and clinical studies for their potential to detect, diagnose, and treat various diseases. Their distinct material and size-dependent properties, differing from organic nanoparticles, have garnered significant attention [82]. MNPs-based nanomedicines that have received approval from the Food and Drug Administration (FDA) and are currently being utilized in clinical settings have displayed enhanced bioavailability and efficacy in drug delivery systems. By enhancing targeted delivery and promoting active cellular uptake, these systems reduce adverse effects. Optimizing the size, shape, surface chemistry, and doping techniques of MNPs allows them to degrade quickly under specific physiological conditions and be efficiently metabolized, minimizing harm to healthy tissues [83]. Table 3 provides examples of FDA-approved MNPs used in clinical trials.

**Table 3.** FDA approved metal nanoparticles.

Name	Nanomaterial	Indication	Clinical Trial Identifier (Phases)
Nanotherm®	Iron oxide nanoparticles	Brain tumor	Approved by the EMA in 2010
Magnablate®	Iron oxide nanoparticles	Prostate cancer	NCT02033447 (Early Phase I): Completed
Nanotherm®	Iron oxide nanoparticles	Prostate carcinoma	NCT05010759: Still recruiting (Phase not applicable)
NU-0129®	Spherical gold nanoparticle conjugated with siRNA oligonucleotides	Glioblastoma multiforme or Gliosarcoma Treatment	NCT03020017: Completed
	Silver nanoparticle/calcium hydroxide	Postoperative pain	NCT03692286 (Completed), NCT04213716 (Completed)

The table highlights different nanomaterials undergoing clinical trials for various medical uses. Iron oxide nanoparticles were tested in a Phase I trial (NCT02033447) for prostate cancer, which has since been completed. Spherical gold nanoparticles have been examined for their potential in treating recurrent glioblastoma or gliosarcoma in patients undergoing surgery, with the study concluding under trial number NCT03020017. Additionally, silver nanoparticles combined with calcium hydroxide were assessed in two trials (NCT03692286, NCT04213716) for their effectiveness in managing postoperative pain, both of which have been completed.

Silver nanoparticles have a well-established history in clinical settings and were among the first nanomedicines to receive approval in the 1990s [148]. Gold nanoparticles (AuNPs) are employed in bioimaging and biosensing for pathogen detection, in targeted drug delivery for anticancer therapy and photothermal treatment, and as antimicrobial agents against various pathogens (Patil et al., 2022). Palladium nanoparticles are acknowledged for their controlled drug-release capabilities and anti-cancer properties, rendering them valuable in drug-delivery systems [148]. The biomedical potential of platinum nanoparticles is currently under exploration, particularly regarding their anti-cancer capabilities and suitability for controlled drug delivery [148].

Copper and copper oxide nanoparticles have gained considerable interest in biomedicine due to their benefits, including improved drug stability, biodistribution, therapeutic index, and targeted delivery [150]. However, challenges such as biocompatibility and controlled drug release hinder their clinical application. Woźniak-Budych et al. highlight the importance of comprehensive toxicity assessments and developing strategies to address these issues, ensuring the safe and effective use of copper-based nanomaterials in clinical settings [150].

Zinc oxide nanoparticles (ZnO NPs) are widely utilized in biomedical applications, including antibacterial treatments, anticancer therapies, drug delivery, and biosensing. Youssef et al. highlight the advantages of green synthesis methods, which use plants or microbes, offering an eco-friendly and cost-effective alternative. ZnO nanoparticles generate ROS that trigger apoptosis, making them especially effective in targeting cancer cells and pathogens, and thereby playing a vital role in contemporary medical treatments. Additionally, titanium dioxide nanoparticles are being researched for their use in drug delivery, especially in cancer treatments, due to their stability and minimal toxicity [148].

Metallic nanoparticles have revolutionized drug delivery systems by enhancing stability, prolonging the half-life of drug carriers, and facilitating precise targeted delivery. These advancements enable passive and active targeting methods, significantly improving therapeutic outcomes. Moreover, the development of green synthesis methods for metallic nanoparticles provides eco-friendly and cost-effective alternatives to conventional chemical and physical fabrication techniques [147].

**Figure 4.**
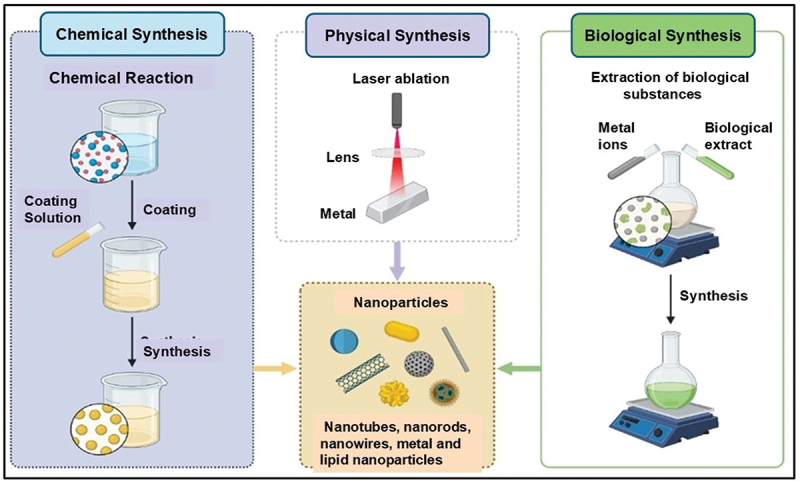
The various approaches employed in the synthesis of metal nanoparticles. This figure outlines the diverse techniques used for synthesizing metal nanoparticles. These include physical approaches like laser ablation and thermal decomposition, chemical processes such as chemical reduction and sol-gel methods, and biological approaches utilizing plant extracts, bacteria, and fungi. Each method has its benefits; physical and chemical techniques provide precise control over nanoparticle size and shape, while biological methods offer advantages like environmental sustainability and biocompatibility. These synthesis strategies are crucial for developing nanoparticles for various fields, including medicine, electronics, and environmental applications. created with Biorender.com.

There are several methods for synthesizing metal nanoparticles, including chemical reduction, microemulsion, thermal decomposition, and microwave-assisted techniques (Figure 4) [151]. These processes often rely on strong chemical agents like dimethylformamide and sodium borohydride, which function as reducing and capping agents under high-temperature and vacuum conditions. However, such methods present environmental risks, as the hazardous chemicals and waste byproducts can harm soil, water, and human health [4].

Increasingly, researchers are turning to environmentally sustainable practices for synthesizing nanoparticles by utilizing plant-based materials such as roots, fruits, leaves, stems, and flowers. These materials are favored for their eco-friendliness, simplicity, speed, and stability [4,146–148]. In photo-nanotechnology, the synthesis of nanoparticles often utilizes water as a solvent, ensuring a nontoxic and straightforward process [5,6]. Plant extracts, rich in proteins, amino acids, vitamins, and secondary metabolites, act as essential reducing, capping, and stabilizing agents in the fabrication of metal nanoparticles [7,8].

This environmentally conscious approach mitigates the hazards associated with chemical processes and aligns with the 12 principles of green chemistry, emphasizing the production of low-toxicity materials [147,148]. Green synthesis methodologies are prevalent in nanomedicine and nano drug delivery systems, employing plant-derived compounds traditionally utilized in various medical treatments. Notably, approximately 25% of medications stem from natural sources, underscoring the pivotal role of plant-based compounds in drug discovery and development owing to their varied chemical properties, biological efficacy, cost-effectiveness, minimal side effects, and diminished toxicity [152,153]. The Introduction of metal nanoparticles encapsulating BBR exhibits significant potential for advancing drug delivery research. This pioneering approach holds promise for more precise and efficient therapeutic strategies. Subsequent sections will explore the latest advancements and insights regarding the utilization of BBR-loaded metal nanoparticles in drug delivery systems.

## 6. Metal-based nanoplatforms for berberine

Recent advancements in nanoscience and technology have spurred the development of innovative techniques to enhance the solubility of BBR, such as solid dispersion, particle size reduction, and nanoparticle encapsulation [146–148]. Nanoparticle-based delivery systems have been developed to overcome the challenges of oral BBR administration and improve its therapeutic effectiveness. Various nanocarriers, including polymeric, lipid-based, and those crafted from silver and gold, are utilized to deliver BBR [146]. Metallic nanocarriers stand out in drug delivery applications due to their distinctive characteristics, including a large surface area, customizable pore size, high pore volume, and adaptable surface properties [147]. These sophisticated carriers facilitate controlled drug release at specific sites, enhancing effectiveness and reducing toxicity compared to conventional delivery methods [148].

Metallic nanoparticles enhance BBR’s bioavailability and therapeutic efficacy through multiple interconnected mechanisms. First, they improve BBR’s solubility by encapsulating the hydrophobic drug within hydrophilic coatings or matrices, ensuring better dispersion in aqueous environments. This prevents premature aggregation and degradation, leading to enhanced absorption in biological systems. Additionally, metal-based nanoplatforms provide a controlled and sustained drug release profile, preventing rapid metabolism and clearance, thereby prolonging berberine’s therapeutic action.

Another critical aspect is targeted drug delivery. Surface modifications, such as polyethylene glycol (PEG) or ligand functionalization, facilitate nanoparticle-mediated transport across biological barriers, improving cellular uptake. Metallic nanoparticles, typically made from metals such as gold, silver, iron, and zinc (Table 4) [147], can be guided to specific tissues using external stimuli, such as magnetic fields or laser irradiation, enhancing site-specific delivery. Moreover, they protect berberine from enzymatic degradation, ensuring its stability in systemic circulation. Collectively, these properties enable metallic nanoparticles to significantly improve the pharmacokinetics and therapeutic potential of berberine, making them promising candidates for advanced drug delivery applications [148]. Additionally, these nanoparticles boost the effectiveness of anticancer drugs by enabling photothermal excitation and allowing for the tracking of their distribution within the body using light [4].

**Table 4.** Summary of metallic nanoparticles used in Berberine Delivery: methods of synthesis, key properties, and therapeutic potential.

Nanoparticle Type	Synthesis Method	Size (nm)	Surface Modification	Therapeutic Application	Reference
Gold (AuNPs)	Green synthesis, chemical reduction	10–100	PEGylation, ligand conjugation	Cancer therapy, drug delivery	[154]
Silver (AgNPs)	Biopolymer-mediated, microwave synthesis	10–150	Protein coating, surface oxidation	Antimicrobial, anticancer	[155]
Zinc Oxide (ZnO)	Hydrothermal, sol-gel method	20–200	Chitosan, polymer coating	Antibacterial, drug carrier	[156]
Selenium (SeNPs)	Biological reduction, electrochemical	50–150	Biopolymer conjugation	Antioxidant, anticancer	[157]
Iron Oxide (Fe3O4)	Co-precipitation, thermal decomposition	10–300	Carboxylation, magnetic targeting	MRI contrast, drug delivery	[154]

The table provides an overview of different metallic nanoparticles, their synthesis approaches, typical sizes, surface modifications, and therapeutic uses, particularly in the contexts of cancer therapy, antimicrobial treatments, and drug delivery. References are cited for further details on each nanoparticle type and its application.

### 6.1. Gold nanoparticles

In the 19^th^ century, Michael Faraday was the first to introduce gold nanoparticles, distinguished by their unique properties such as surface plasmon resonance (SPR), the capacity to form bonds with amine and thiol groups, and the potential for surface modification, rendering them highly suitable for biomedical applications. Gold nanoparticles fulfil various functions including serving as carriers for pharmaceuticals, contrast agents, photochemical agents, and radiosensitizers, exhibiting significant potential in cancer therapy [148].

Souza et al. improved BBR delivery by combining gold nanoparticles with gellan gum and polyvinyl alcohol, resulting in a highly efficient drug delivery system [4]. Similarly, Pandey et al. used gold nanoparticles conjugated with folic acid to target berberine to solid tumors, enhancing its anticancer activity against HeLa cells. This approach achieved high BBR loading and selective cytotoxicity, showing reduced toxicity to normal cells and effective drug release in acidic environments [5].

Chui et al. studied a gold nanoparticle-collagen nanocarrier with BBR (Au-Col-BB) and found it more effective in inducing apoptosis and reducing tumor growth in Her-2 breast cancer cells compared to non-cancerous cells. The research suggests that Au-Col-BB could be a promising treatment for breast cancer [6,158].

### 6.2. Silver nanoparticles

Silver nanoparticles are highly valued for their distinctive physical and chemical properties, such as their unique optical and thermal attributes, as well as their high electrical conductivity. These attributes make them extremely valuable in a myriad of biomedical applications [115]. They have also been shown to enhance the anticancer effects of some drugs [147].

Dziedzic and colleagues studied the impact of AgNPs on oral cancer cells, both alone and combined with BBR. While AgNPs on their own had antiproliferative effects, the addition of BBR reduced this effect, likely due to electrostatic interactions between the positively charged BBR and negatively charged AgNPs. This suggested that while AgNPs alone reduced cancer cell viability, the combination with BBR weakened their effectiveness [159]. Bhanumathi et al. further demonstrated that BBR-loaded AgNPs had dose-dependent cytotoxic effects on MCF-7 and MDA-MB-231 breast cancer cells [160].

### 6.3. Zinc oxide nanoparticles

Recent advances in nanotechnology have led to the creation of new hybrid nanoparticle systems combining organic and inorganic materials for better cancer treatment. In a 2018 study, Kim, Lee, and Cho developed BBR and zinc oxide (ZnO) nanoparticles to treat lung adenocarcinoma. These BBR-ZnO nanoparticles were created using a simple blending method without extra additives, making them easy to produce on a large scale. The particles, sized between 200–300 nm, were mostly made of ZnO, with a 39:61 ratio of BBR to ZnO. The study showed that these nanoparticles had strong antiproliferative effects on A549 lung cancer cells due to their combined chemical and heat-based (photothermal) effects. The nanoparticles’ ability to kill cancer cells using chemical and thermal methods offers a promising lung cancer treatment. Tests confirmed the structural stability of the ZnO and the amorphization of BBR within the nanoparticles. Additionally, safety tests in rats showed no severe liver, kidney, or blood toxicity after intravenous use, suggesting that BBR-ZnO nanoparticles can be a safe and effective treatment for lung cancer through chemo-photothermal therapy [161].

### 6.4. Selenium nanoparticles

Othman et al. conducted a study where they created and tested selenium nanoparticles (SeNPs) loaded with BBR to explore their cancer-fighting abilities. In experiments with Swiss albino mice injected with tumor cells, the SeNPs with BBR improved survival rates, reduced tumor size, and decreased body weight compared to the control group. These nanoparticles also lowered oxidative stress and nitric oxide levels while increasing glutathione, showing their effectiveness. The study also found that BBR-loaded SeNPs triggered apoptosis in tumor cells, suggesting their potential as a chemotherapy treatment using eco-friendly methods [10].

In a study conducted by Khaled et al., the effects of BBR-SeNPs were assessed on human liver cancer cells (HepG2) and normal mouse liver cells. The findings revealed that these nanoparticles exhibited strong antitumor activity against HepG2 cells, effectively inducing apoptosis and causing cell cycle arrest. BBR-SeNPs were found to be more effective than BBR-loaded silver nanoparticles (BBR-AgNPs), likely due to selenium’s intrinsic anticancer properties [156]. Additionally, Othman et al. explored the anticancer potential of green-synthesized BBR-SeNPs against Ehrlich solid tumors. Their results showed that treatment with BBR-SeNPs significantly improved survival rates and reduced tumor size, further emphasizing the potential of BBR-SeNPs as a promising anticancer agent [148].

### 6.5. Iron-oxide nanoparticles

Iron-oxide nanoparticles combined with BBR have shown significant potential in targeting hypoxic tumors, which are notoriously difficult to treat due to their resistance to conventional therapies. In a study by Sreeja and Nair, a complex consisting of iron-oxide nanoparticles, berberine, and the hypoxic cell sensitizer, sanazole (NP-BBN-SAN), was developed and tested in a mouse model bearing solid tumors [162]. The NP-BBN-SAN complexes were carefully characterized using techniques such as FTIR, XRD, TEM, and nano-size analysis. When orally administered to Swiss albino mice with solid tumors, the NP-BBN-SAN complex effectively reduced tumor volume. The study revealed that the complex induced significant DNA damage in tumor cells downregulated the transcription of hypoxia-related genes (hif-1α, VEGF, Akt, and bcl2), and upregulated pro-apoptotic genes like Bax and caspases. These molecular changes were corroborated by histopathological analyses, which showed the extensive therapeutic specificity of the NP-BBN-SAN complex in targeting tumor tissues while sparing healthy tissues like the liver and kidney [162].

### 6.6. Magnetic nanoparticles

The functionalization of magnetic nanoparticles with BBR has been extensively studied to create advanced drug delivery systems. As reported, carboxymethyl chitosan-coated Fe₃O₄ nanoparticles were successfully synthesized and loaded with BBR, demonstrating efficient drug-loading capacity and biocompatibility [154]. These nanoparticles were specifically engineered to ensure stability in physiological conditions and a controlled release profile, thereby addressing the solubility and bioavailability issues associated with BBR.

Janus magnetic mesoporous silica nanoparticles have also been employed as carriers for BBR, offering a unique dual-functional design. These nanoparticles displayed excellent biocompatibility and pH-responsive drug release, a crucial feature for achieving site-specific delivery in tumor microenvironments. Furthermore, the incorporation of a magnetic field enhanced the localization and therapeutic efficacy of BBR-loaded nanoparticles, particularly in hepatocellular carcinoma models [163–165].

In cancer therapy, BBR-loaded magnetic nanoparticles have demonstrated notable potential. Studies have shown that these systems enhance the cytotoxicity of BBR against gastric cancer cells, attributed to improved cellular uptake and targeted delivery capabilities [154]. Research on breast cancer treatment has similarly revealed that these nanoparticles substantially reduce tumor growth by delivering BBR directly to the cancer site while minimizing systemic toxicity [166]. Beyond cancer, magnetic nanoparticles improve the stability and bioavailability of BBR and enable external manipulation via magnetic fields, allowing for advanced therapeutic approaches such as magnetically guided drug delivery and hyperthermia [167].

## 7. Challenges and future perspectives

As our literature research has indicated, the use of MNPs as carriers for BBR is relatively limited, and this can be attributed to several significant challenges. The synthesis of BBR-loaded MNPs presents both hurdles and opportunities that are crucial for advancing research in this area.

One primary challenge associated with MNPs of BBR is the precise control over the size, shape, and surface properties of the nanoparticles. Fine-tuning the reaction conditions to achieve consistency and reproducibility is difficult, and even minor variations can significantly impact the nanoparticles’ performance. Ensuring the stability and sustained effectiveness of BBR-based metallic nanoparticles in complex biological environments is crucial. A major challenge lies in maintaining their stability when exposed to biological fluids, pH fluctuations, and other in vivo conditions. Current research is focused on stabilizing and functionalizing these nanoparticles to enhance their biocompatibility and therapeutic efficacy.

Scalability and cost-effectiveness are also major obstacles in the production of metallic nanoparticles. Although green synthesis methods offer environmentally friendly alternatives to traditional chemical processes, scaling up these methods while maintaining consistent quality and efficiency is critical. The scalability of metal-based nanocarriers remains an issue due to batch-to-batch variability, high production costs, and the need for specialized equipment. Novel synthesis approaches, such as green chemistry techniques and continuous flow synthesis, may enhance large-scale manufacturability.

In addition to these synthesis and application challenges, the potential toxicity of metal-based nanoparticles poses significant concerns. The unique physicochemical properties that make MNPs effective in biomedical applications, such as high surface area and reactivity, also contribute to their potential toxicity. The generation of reactive oxygen species (ROS) by MNPs can lead to oxidative stress, damaging cellular components like DNA, proteins, and lipids, which may result in cell death. Furthermore, the interaction of MNPs with cellular membranes can disrupt membrane integrity, leading to altered permeability and loss of cellular homeostasis. These toxic effects are further complicated by the nanoparticles’ ability to cross biological barriers, leading to unintended accumulation in non-target tissues such as the liver, spleen, and kidneys, potentially causing organ toxicity over time. Additionally, one major concern is their controlled and sustained drug release, as the release kinetics can be affected by nanoparticle aggregation, dissolution rates, and stability under physiological conditions. Again, their therapeutic efficacy varies depending on the nanoparticle type and surface modifications, necessitating extensive optimization for different disease models. The interaction of metal nanoparticles with biological fluids and proteins can lead to unpredictable pharmacokinetics, altering drug distribution and reducing therapeutic targeting. Moreover, the potential immunogenicity and inflammatory responses triggered by some metallic nanoparticles can lead to adverse side effects, limiting their long-term clinical use.

The approval of metal-based nanocarriers by regulatory agencies such as the FDA and EMA is complex due to concerns regarding their long-term toxicity, environmental impact, and reproducibility. Standardized protocols for toxicity assessment and clinical validation are needed to streamline regulatory approval.

Despite these challenges, the prospects for synthesizing metallic nanoparticles loaded with BBR remain promising, supported by continuous advancements in nanotechnology and biomedicine. Recent studies suggest that BBR-loaded metallic nanoparticles have substantial potential for various biomedical applications, including targeted drug delivery, diagnostic imaging, and cancer therapy. Researchers are increasingly interested in leveraging BBR’s unique properties and innovative synthesis techniques to overcome current limitations and explore new possibilities in nanomedicine.

Future research should focus on advancing functionalization techniques, such as ligand attachment, polymer coatings, and bio-inspired modifications, to improve the biocompatibility and targeting efficiency of metal-based nanoplatforms. Functionalized nanoparticles enhance cellular uptake and site-specific drug delivery, reducing systemic toxicity and off-target effects. The integration of metal-based nanoparticles with polymeric or lipid-based carriers presents an opportunity to leverage the advantages of multiple nanoplatforms. Hybrid nanoparticles can optimize drug release kinetics, reduce cytotoxicity, and improve biodistribution. For instance, lipid-coated metal nanoparticles combine the stability of metals with the biocompatibility of lipids, making them ideal candidates for drug delivery applications.

Moving from preclinical research to clinical applications requires extensive pharmacokinetic and pharmacodynamic studies. Regulatory approval processes demand thorough investigations into the safety, efficacy, and long-term stability of metal-based nanoplatforms. Collaborations between academia, industry, and regulatory agencies are essential to streamline the transition from laboratory research to real-world medical applications. A major challenge for metal-based nanoparticles is their long-term accumulation in biological tissues. Prolonged retention in the liver, spleen, and kidneys can lead to toxicity concerns. Future research should explore biodegradable or excretable metallic nanostructures that can be safely eliminated from the body. Strategies such as enzyme-responsive nanoparticles or organic-metal hybrid systems may enhance clearance while maintaining therapeutic efficiency. Furthermore, tailoring metal-based nanoparticle formulations for specific patient populations and disease conditions through advanced computational modeling and precision medicine strategies could optimize therapeutic outcomes. Machine learning algorithms and artificial intelligence-driven designs may enable the development of highly efficient, patient-specific drug delivery systems that enhance treatment effectiveness while minimizing adverse effects.

By tackling these challenges, future works can pave the way for advanced BBR-based nanotherapeutics that are both effective and safe for clinical use.

## 8. Conclusion

The development of metallic nanoparticles loaded with berberine (BBR) presents both significant challenges and exciting opportunities in nanomedicine. Precise control over nanoparticle properties and scalable production remains key obstacles; however, continuous progress in nanotechnology is paving the way for solutions. The distinctive physicochemical characteristics and multifunctionality of BBR-loaded metallic nanoparticles hold great potential for biomedical applications, including targeted drug delivery, diagnostic imaging, and cancer therapy.

Future advancements will require interdisciplinary collaboration and the exploration of innovative synthesis techniques to facilitate the transition from fundamental research to clinical applications. Enhancing synthesis methodologies and optimizing nanoparticle formulations are essential steps toward their effective clinical translation. By addressing these challenges and leveraging emerging opportunities, researchers can drive the development of the next generation nanotherapeutics based on BBR, ultimately contributing to improved healthcare outcomes and patient well-being.

## Supplementary Material

Supplemental Material

## Article highlights

- The review provides a comprehensive analysis of the current progress, challenges, and prospects of metal-based nanoplatforms in enhancing berberine’s biomedical applications.
- Berberine (BBR) is a bioactive isoquinoline alkaloid with a wide range of therapeutic potential, suffers from limited clinical uses due to its poor solubility, low bioavailability, and quick metabolism.
- The therapeutic efficacy of berberine has been increased by recent developments in nanocarrier systems, which have improved cellular uptake, extended circulation time, and site-specific drug delivery.
- By improving berberine’s stability, controlled drug release, and targeted delivery, metal-based nanoplatforms present encouraging alternatives.
- The effectiveness of metal-based nanocarriers in transporting berberine and their bioactivity are largely determined by their physicochemical characteristics.
- Currently, challenges with synthesis, reproducibility and regulatory obstacles limit the large-scale production and application of metal-based nanocarriers.
- To overcome the limitations, future research should concentrate on improving hybrid nanocarrier systems, functionalization methods, and nanoparticle formulations.
- Before clinical translation, thorough research on biocompatibility and long-term safety is necessary due to toxicity concerns, which continue to be a major obstacle.

## (CRediT) author statement

Conceptualization, I.B., P.S., H.A., and B.P.G.; writing – original draft preparation, I.B. and P.S.; writing – review and editing, P.S., H.A., and B.P.G.; supervision, P.S. and B.P.G.
