# Supplementary figures and images for: Metal-based nanoplatforms for enhancing the biomedical applications of berberine: current progress and future directions
Source: Nanomedicine (Lond). 2025 Mar 20;20(8):851–68. doi: 10.1080/17435889.2025.2480051 (PMC11999359; doi:10.1080/17435889.2025.2480051)

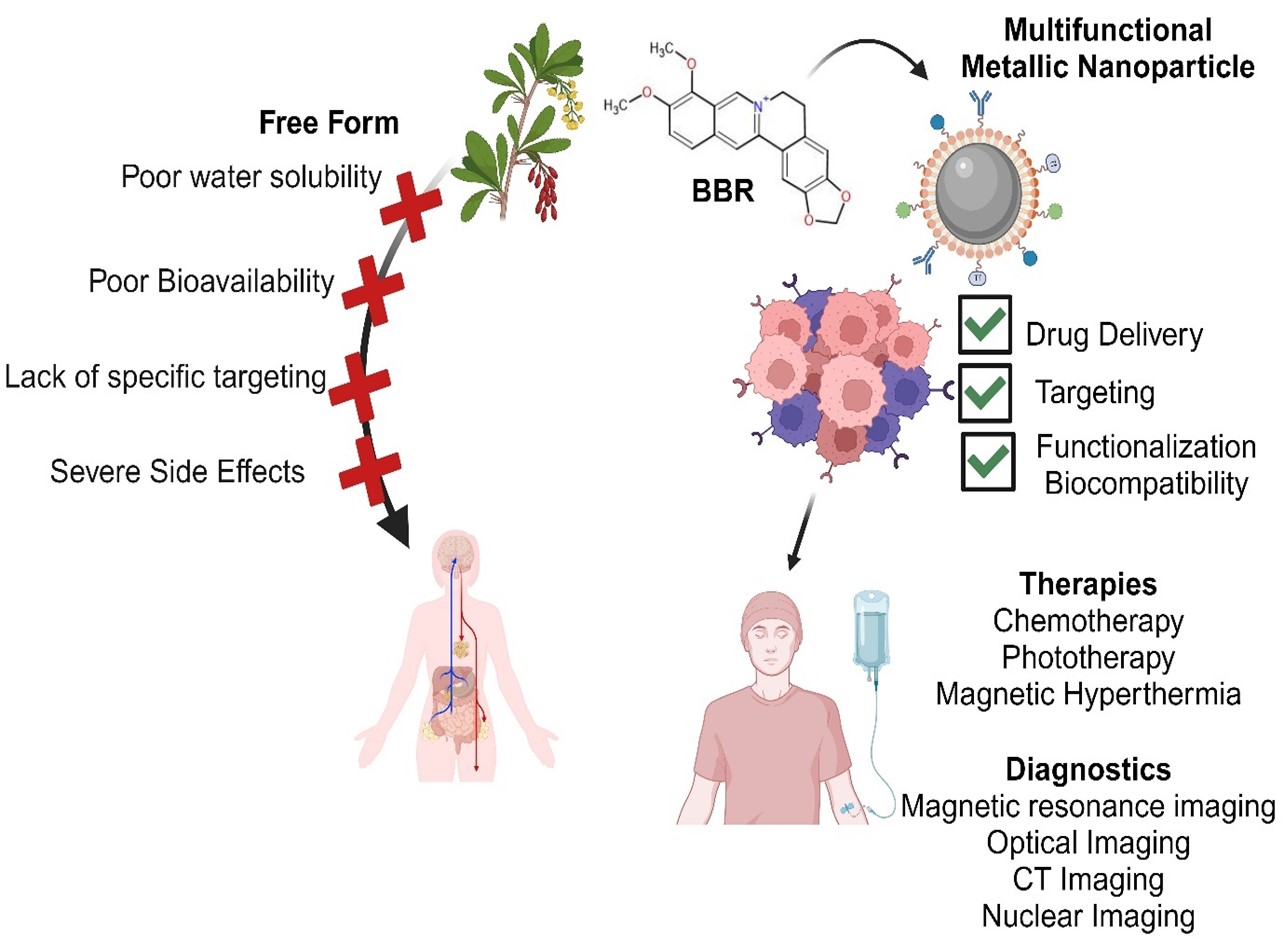

Supplement: Supplemental Material [file INNM_A_2480051_SM2064.jpg]
